# The role of growth differentiation factor 15 in the pathogenesis of primary myelofibrosis

**DOI:** 10.1002/cam4.502

**Published:** 2015-08-15

**Authors:** Tatsuki Uchiyama, Hiroshi Kawabata, Yasuo Miura, Satoshi Yoshioka, Masaki Iwasa, Hisayuki Yao, Soichiro Sakamoto, Masakazu Fujimoto, Hironori Haga, Norimitsu Kadowaki, Taira Maekawa, Akifumi Takaori-Kondo

**Affiliations:** 1 Department of Hematology and Oncology Graduate School of Medicine, Kyoto UniversityKyoto, Japan; 2 Department of Transfusion Medicine and Cell Therapy, Kyoto University HospitalKyoto, Japan; 3 Division of Gastroenterology and Hematology, Shiga University of Medical ScienceOtsu, Japan; 4 Department of Diagnostic Pathology, Kyoto University HospitalKyoto, Japan

**Keywords:** Growth differentiation factor 15, osteoblastic differentiation, primary myelofibrosis

## Abstract

Growth differentiation factor 15 (GDF15) is a pleiotropic cytokine that belongs to the transforming growth factor-*β* superfamily. Elevated serum concentrations of this cytokine have been reported in patients with various malignancies. To assess the potential roles of GDF15 in hematologic malignancies, we measured its serum levels in patients with these diseases. We found that serum GDF15 levels were elevated in almost all these patients, particularly in patients with primary myelofibrosis (PMF). Immunohistochemical staining of bone marrow (BM) specimens revealed that GDF15 was strongly expressed by megakaryocytes, which may be sources of increased serum GDF15 in PMF patients. Therefore, we further assessed the contribution of GDF15 to the pathogenesis of PMF. Recombinant human (rh) GDF15 enhanced the growth of human BM mesenchymal stromal cells (BM-MSCs), and it enhanced the potential of these cells to support human hematopoietic progenitor cell growth in a co-culture system. rhGDF15 enhanced the growth of human primary fibroblasts, but it did not affect their expression of profibrotic genes. rhGDF15 induced osteoblastic differentiation of BM-MSCs in vitro, and pretreatment of BM-MSCs with rGDF15 enhanced the induction of bone formation in a xenograft mouse model. These results suggest that serum levels of GDF15 in PMF are elevated, that megakaryocytes are sources of this cytokine in BM, and that GDF15 may modulate the pathogenesis of PMF by enhancing proliferation and promoting osteogenic differentiation of BM-MSCs.

## Introduction

Growth differentiation factor 15 (GDF15), also known as PL74, macrophage inhibitory cytokine-1 (MIC-1), placental bone morphogenetic protein (PLAB), prostate-derived factor (PDF), placental transforming growth factor-*β* (PTGFB), and nonsteroidal anti-inflammatory drug activated gene-1 (NAG-1), is a pleiotropic cytokine belonging to the bone morphogenetic protein (BMP) subfamily of the transforming growth factor-*β* (TGF-*β*) superfamily 1–7. Various types of cells express GDF15 in response to cellular stresses such as inflammation and ischemia, although its steady-state expression is almost completely restricted to the placenta 5. In cells, GDF15 is initially translated as a 62-kD proprotein, and it is then processed by a furin-like protease to produce 25-kD mature peptides that form a disulfide-linked homodimer 8. Specific receptors and signaling pathways for GDF15 have still not been determined; however, roles in stress responses, inflammation, tissue repair after acute injury, energy homeostasis, systemic iron metabolism, and malignancy have been suggested 9,10.

Serum concentrations of GDF15 increase during pregnancy as a result of strong expression in the placenta 5. In pathological conditions, elevated serum GDF15 levels have been identified as an independent risk factor for acute coronary heart diseases 11,12. In addition, the serum GDF15 concentration is notably increased in patients with glioma, prostate, colorectal, or pancreatic cancers, and GDF15 was recently described as one of the 20 biomarkers that best define the malignant phenotype of numerous tumors 9,10,13. However, its biological roles in cancers are complex and poorly understood; some studies have documented its protumorigenic activities, whereas others have demonstrated its antitumorigenic activities, which appear to be dependent on the types and stages of cancers 9.

In hematopoietic diseases, serum GDF15 levels are remarkably elevated in patients with dyserythropoietic disorders such as *β*-thalassemia, congenital dyserythropoietic anemia type I, and refractory anemia with ring sideroblasts, a subtype of myelodysplastic syndromes (MDS) 14–17. Common features of these diseases include systemic iron overload and ineffective erythropoiesis, and the major source of the serum GDF15 in these diseases is assumed to be erythroid cells 14. Recently, serum GDF15 levels in polycythemia vera (PV) and essential thrombocythemia (ET), which are two types of myeloproliferative neoplasm (MPN), were shown to be increased in comparison with the levels in healthy controls 18. In addition, serum levels of GDF15 were reported to be elevated in patients with multiple myeloma (MM), and bone marrow (BM) mesenchymal stromal cells (MSCs) from these patients were shown to express high levels of GDF15 19–22. It was speculated that GDF15 secreted from these cells may play roles in the pathogenesis of MM by enhancing the tumor-initiation and self-renewal potential of MM cells 21.

In this study, we evaluated serum levels of GDF15 in patients with various hematologic diseases. We found that patients with primary myelofibrosis (PMF) showed very high serum GDF15 levels, which prompted us to further investigate the source of GDF15 in the BM, and to assess the contribution of GDF15 to the pathogenesis of PMF.

## Materials and Methods

### Reagents

Diosgenin and pholbol-13 myristate-12 acetate (PMA) were purchased from Sigma-Aldrich (St. Louis, MO). Hemin was obtained from Alfa Aesar (Ward Hill, MA). Recombinant human growth differentiation factor 15 (rhGDF15) was purchased from R&D Systems (Minneapolis, MN). Recombinant human transforming growth factor-*β*1 (rhTGF-*β*1), stem cell factor (SCF), interleukin-3 (IL-3), and Flt3-ligand (Flt3-L) were purchased from Wako Pure Chemical Industries (Osaka, Japan). Thrombopoietin (TPO) was provided by Kyowa Hakko Kirin (Tokyo, Japan).

### Clinical materials

Serum samples collected from 14 healthy volunteers (median age, 35 years; range, 30–46 years) and 128 adult patients with various hematologic malignancies at Kyoto University Hospital from 2006 to 2015 were used. Diagnoses of these patients were basically based on the WHO Classification of Tumors of Hematopoietic and Lymphoid Tissues 23. Some of the patients had received chemotherapy before serum collection; however, patients in complete remission were excluded. The samples were stored at −80°C until cytokine assays were performed. The BM biopsy samples were obtained from patients with PMF and ET. As normal morphology controls, BM specimens obtained from patients with diffuse large B-cell lymphoma (DLBCL) and gastric cancer without BM involvement were used. Written informed consent for experimental use of their specimens was obtained from all the patients. This study was approved by the ethics committee of Kyoto University Graduate School and the Faculty of Medicine of Kyoto University.

### Determination of serum GDF15 levels

The concentrations of GDF15 in the collected serum samples and culture supernatants were measured by enzyme-linked immunosorbent assay (ELISA) in duplicates by using a human GDF15 DuoSet ELISA Development kit (R&D Systems), according to the instructions of the manufacturer.

### Detection of GDF15 protein in the BM

The BM specimens were fixed in 10% buffered formalin, processed according to conventional histological techniques, embedded in paraffin, and stained with hematoxylin and eosin. Immunohistochemical staining for GDF15 was performed using rabbit polyclonal antibodies against human GDF15 (1:100, anti-human HPA011191; Atlas Antibodies, Stockholm, Sweden) on an automated slide stainer (Ventana XT System Benchmark; Ventana Medical Systems, Tucson, AZ). Images were acquired using a BioZero BZ-8100 microscope (Keyence, Osaka, Japan).

### Cell lines, culture conditions and in vitro cell differentiation and proliferation assays

HEL (JAK2 V617F positive human erythroleukemia) cells were cultured in RPMI 1640 (Nacalai Tesque, Kyoto, Japan) supplemented with 10% fetal bovine serum (FBS; Sigma-Aldrich) and 100 U/mL penicillin and 100 *μ*g/mL streptomycin (Nacalai Tesque) in a humidified 5% CO_2_ incubator at 37°C. To induce megakaryocytic differentiation, HEL cells were grown in the presence of 10 *μ*mol/L diosgenin or 10 nmol/L PMA. Additionally, to induce erythroid differentiation, the cells were treated with 50 *μ*mol/L hemin. After 96 h of culture, approximately 3 × 10^4^ cells were collected onto a glass slide by centrifugation for 4 min at 600*g* using Cytospin3 (Thermo Shandon, Pittsburgh, PA). After air-drying, the slides were stained with May-Grunwald-Giemsa (Sigma-Aldrich), and observed using light microscopy.

### Hematopoietic progenitor cell expansion assay

Human bone marrow mesenchymal stromal cells (BM-MSCs) were purchased from AllCells (Emeryville, CA) and cultured in advanced-minimal essential medium (Thermo Fisher Scientific Inc., Waltham, MA) supplemented with 5% fetal bovine serum (FBS, Thermo Fisher Scientific), 100 *μ*mol/L ascorbic acid (Wako Pure Chemical Industries), 2 mmol/L l-glutamine, 100 U/mL penicillin, and 100 *μ*g/mL streptomycin (all from Gibco, Carlsbad, CA) as described previously^24^–^27^. Normal human BM mononuclear cells were separated from BM aspirates (AllCells) using Ficoll-Hypaque-Plus solution (GE Healthcare, Tokyo, Japan). CD34^+^HPCs (Hematopoietic progenitor cell) were isolated from BM mononuclear cells by using anti-CD34 immunomagnetic microbeads (Miltenyi Biotec, Bergisch Gladbach, Germany). For in vitro HPC expansion assays, culture-expanded human BM-MSCs (2 × 10^4^ cells/well) were seeded in a 24-well culture plate. Then, CD34^+^ HPCs isolated from normal human BM mononuclear cells were applied (0.6 × 10^3^ cells/well) and co-cultured in StemSpan Serum-free Expansion Medium (SFEM; StemCell Technologies, Vancouver, Canada) supplemented with 100 ng/mL SCF, 100 ng/mL Flt3-L, 20 ng/mL IL-3, and 50 ng/mL TPO. After 10 days of co-culture, the number and surface marker expression of the expanded hematopoietic cells were determined by flow cytometry with fluorescence-conjugated antibodies. Fluorescein isothiocyanate (FITC)-conjugated anti-human CD45 and phycoerythrin (PE)-conjugated mouse anti-human CD34 antibodies were purchased from BD Pharmingen (San Diego, CA), and allophycocyanin (APC)-conjugated mouse antibody against human CD38 was purchased from Bioscience (San Diego, CA). Each analysis included corresponding FITC- or PE-conjugated isotype controls. Dead cells were excluded by staining with propidium iodide. All data were acquired using a FACSCanto II (Becton Dickinson, San Jose, CA), and 10,000 events per sample were collected. Acquired data were subsequently analyzed with FlowJo software (Tree Star, Ashland, OR).

### Culture conditions of normal human primary dermal fibroblasts

Normal human primary dermal fibroblasts (NHDF) were purchased from Lonza (Walkersville, MD) and maintained in fibroblast growth medium-2 (FGM-2) BulletKit (CC-3132, Lonza) in a humidified 5% CO_2_ incubator at 37°C. For proliferation assays, NHDFs were plated at a density of 2 × 10^4^ cells/well in 6-well plates with FGM-2 in five replicates. After 24 h of incubation, the culture medium was replaced with Dulbecco's modified Eagle's medium (DMEM, Nacalai Tesque) containing 1% FBS in the presence of 30 ng/mL rhGDF15 or 2 ng/mL rhTGF-*β*1 in triplicate. Then, after an additional 48, 72, or 96 h of culture, the cells were trypsinized, resuspended, and counted in a hemocytometer by using Trypan blue exclusion. For growth inhibition assays, NHDFs were treated with either 1 *μ*g/mL anti-GDF15 neutralizing mAb (clone 147627, MAB957, R&D Systems) or the same concentration of control isotype-matched mouse IgG 28. For gene expression analysis, NHDF cells were treated with 50 ng/mL rhGDF15 or 2 ng/mL rhTGF-*β*1 in serum-free DMEM for 3 days.

### Evaluation of proliferation and differentiation of human BM-MSCs in vitro

Human BM-MSCs that had just been thawed were seeded at a density of 2 × 10^4^ cells/well in 6-well plates with the medium in five replicates. After 24 h of incubation, the cell culture medium was replaced with MSC growth medium with or without rhGDF15 (30 ng/mL). Adherent cells were detached from the culture dish and viable cells were counted using Trypan blue exclusion after 96, 144, and 192 h without changing the medium.

To analyze the osteogenic differentiation of human BM-MSCs, the cells were seeded at a density of 2 × 10^5^ cells in 8 mL of the medium per 10-cm dish until confluent. The cells were then treated with 30 ng/mL rhGDF15 in the medium, which was replaced every 3–4 days. After 4 weeks of culture, the cells were washed with PBS, fixed in 60% isopropanol, and stained for mineralized deposition with 1% Alizarin Red S dye. The area of the mineralized deposits was quantified using ImageJ software (National Institutes of Health, Bethesda, MD). To analyze adipogenic differentiation, the lipid droplets in the cells were stained with Oil Red O after 2 weeks of culture in MSC growth medium in the presence or absence of 30 ng/mL rhGDF15.

### In vivo bone formation assays

In vivo bone formation assays were performed as previously described 29,30. In brief, a 100-*μ*L cell suspension containing approximately 1 × 10^6^ BM-MSCs was seeded on a hydroxyapatite-poly d,l-lactic-co-glycolic acid composite block type scaffold (HA scaffold; GC Corporation, Tokyo, Japan) and cultured at 37°C in an atmosphere containing 5% CO_2_ for 1 h, and the scaffold was then implanted subcutaneously into the dorsal surface of 7- to 9-week-old nonobese/severe combined immunodeficiency (NOD/SCID) mice. At 10 weeks after implantation, the scaffold was harvested and histological analysis was performed. The area of formed bone in five different fields viewed at ×100 magnification was quantified using ImageJ software as previously described 30. The results are expressed as the ratio of the total bone formation area to the total implant area. All mice were maintained under specific pathogen-free conditions at the Institute of Laboratory Animals, Kyoto University. The animal experiments were approved by the Committee on Animal Research of Kyoto University.

### Immunoblotting analysis

Cell extracts were prepared using Mammalian Protein Extraction Reagent (M-PER; Thermo Fisher Scientific) with complete protease inhibitor cocktail (Thermo Fisher Scientific) according to the manufacturer's instructions. Western blotting was performed by standard methods with anti-phospho p42/p44 MAPK (ERK1/2) (Thr202/Tyr204) (Cell Signaling Technology Inc., Danvers, MA), anti-total ERK1/2, anti-Runx2 (1:1000, ab76956, Abcam, Cambridge, MA), anti-Osterix (SP7) (1:1000, ab22552, Abcam), and anti-*β*-actin (1:1000, A5441, Sigma-Aldrich) antibodies.

### Quantitative real-time PCR analysis

Total RNA was extracted using TRIzol (Thermo Fisher Scientific) and reverse-transcribed into cDNA by using PrimeScript RT Master Mix (Takara Bio, Shiga, Japan), according to the manufacturer's instructions. Real-time PCR was performed with SYBR Premix Ex Taq II (Takara Bio) and with a Thermal Cycler Dice Real Time System TP850 (Takara Bio). The primer sequences used are listed in Supplementary Table S1. The gene expression data were normalized against those of *β*-actin. Each experiment was performed in triplicate cultures, and results were averaged.

### Statistical analyses

Unpaired Student's *t*-test or Fisher's exact test was used for analyses. Data in bar graphs indicate the mean ± SE (*n* = 3), and all experiments were performed in triplicates or duplicates and repeated at least three times. Statistical significance is expressed as follows: **P < 0.01; *P < 0.05; n.s., not significant.

## Results

### Serum GDF15 levels are remarkably elevated in patients with primary myelofibrosis

**Figure 1:**
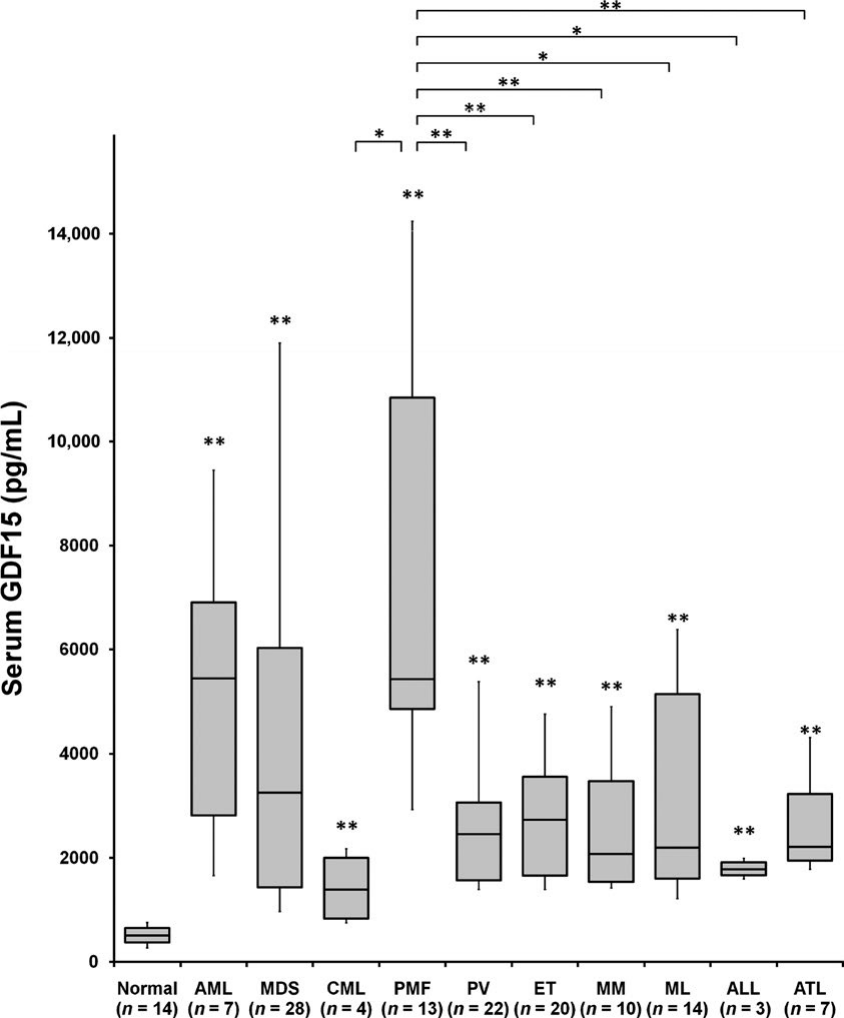
Serum Growth differentiation factor 15 (GDF15 ) levels in patients with various hematological malignancies. Serum concentrations of GDF15 were measured by ELISA in 14 normal subjects and 128 patients with various hematopoietic malignancies. Each individual sample was assayed in duplicate. The box and central bars show interquartile ranges and medians, whereas whiskers on bars above and below the boxes indicate the 90th and 10th percentiles. *P < 0.05; **P < 0.01. Asterisks above the bars indicate significant increases in comparison with normal subjects. AML, acute myelogenous leukemia; MDS, myelodysplastic syndromes; CML, chronic myelogenous leukemia; PMF, primary myelofibrosis; PV, polycythemia vera; ET, essential thrombocythemia; MM, multiple myeloma; ML, malignant lymphoma; ALL, acute lymphoblastic leukemia; ATL, adult T-cell leukemia/lymphoma.

We measured the serum levels of GDF15 in 14 healthy volunteers and in 128 patients with various hematopoietic malignancies, including acute myeloid leukemia (AML; *n* = 7), MDS (*n* = 28), chronic myelogenous leukemia (CML, *n* = 4), PMF (*n* = 13), PV (*n* = 22), ET (*n* = 20), MM (*n* = 10), malignant lymphoma (ML; *n* = 14), acute lymphoblastic leukemia (ALL; *n* = 3), and adult T-cell leukemia (ATL; *n* = 7). The median serum GDF15 levels in all these disease categories were significantly elevated relative to the levels in healthy controls: AML, 4078 pg/mL; MDS, 3249 pg/mL; CML, 1393 pg/mL; PMF, 5425 pg/mL; PV, 2458 pg/mL; ET, 2732 pg/mL; MM, 2074 pg/mL; ML, 2197 pg/mL; ALL, 1780 pg/mL; ATL, 2213 pg/mL; and healthy controls, 505 pg/mL (Fig.1). Among these patients, we noticed that the levels of serum GDF15 were remarkably high in those with PMF, a subtype of MPN, and the levels were significantly higher than the levels of the other types of MPN. PMF patients with advanced disease with more severe anemia tended to show higher serum GDF15 levels (Table S2). Therefore, in the subsequent experiments, we focused on the roles of GDF15 in the pathogenesis of PMF.

### Expression of GDF15 protein in BM predominantly occurs in megakaryocytes

**Figure 2:**
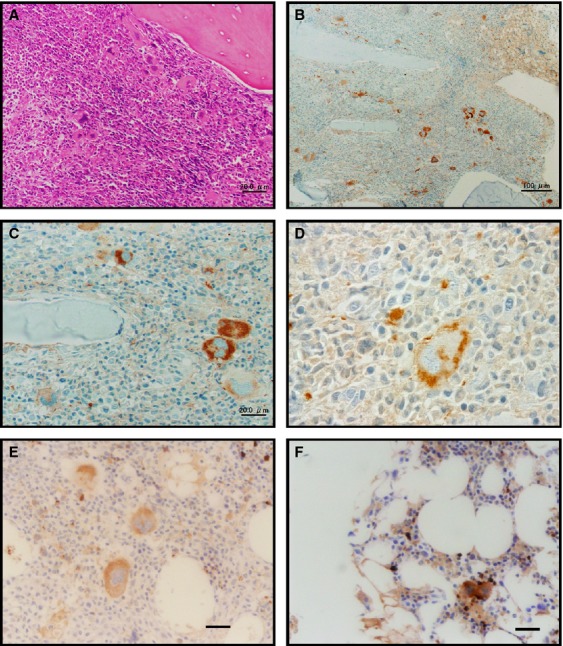
Histological examination of Growth differentiation factor 15 (GDF15) expression in bone marrow (BM) specimens. (A) Hematoxylin and eosin staining and (B–F) immunohistochemical staining for GDF15. (A–D) BM biopsy specimens from patients with primary meylofibrosis and (E) essential thrombocythemia. (F) A BM biopsy specimen with normal morphology obtained from a patient with diffuse large B-cell lymphoma without BM involvement. Original magnification, (A and B) ×100; (C, E, and F) ×400; (D) ×1000.

Previous studies have shown that GDF15 is produced by erythroid cells, macrophages, and MSCs among cell populations in the BM 4,14,20–22. To investigate the source of elevated serum GDF15 in patients with PMF, we examined GDF15 protein expression in paraffin-embedded BM biopsy specimens. Microscopic examination of hematoxylin and eosin-stained specimens of patients with PMF showed increased numbers of megakaryocytes (Fig.2A). Immunohistochemistry (IHC) revealed that GDF15 was expressed predominantly in these megakaryocytes (Fig.2B and C). Apart from these megakaryocytes, which were clearly distinguishable owing to their large sizes, much smaller GDF15-positive cells with round or spindle shape were also observed sporadically (Fig.2C and D). Strong GDF15 expression in megakaryocytes was also observed in specimens of patients with ET (Fig.2E) and specimens of patients with DLBCL without BM involvement (Fig.2F). Similar findings were observed in nondecalcified BM clot sections from patients with ET and localized gastric cancer without BM involvement (Fig. S1).

### GDF15 is upregulated during megakaryocytic differentiation of HEL cells

**Figure 3:**
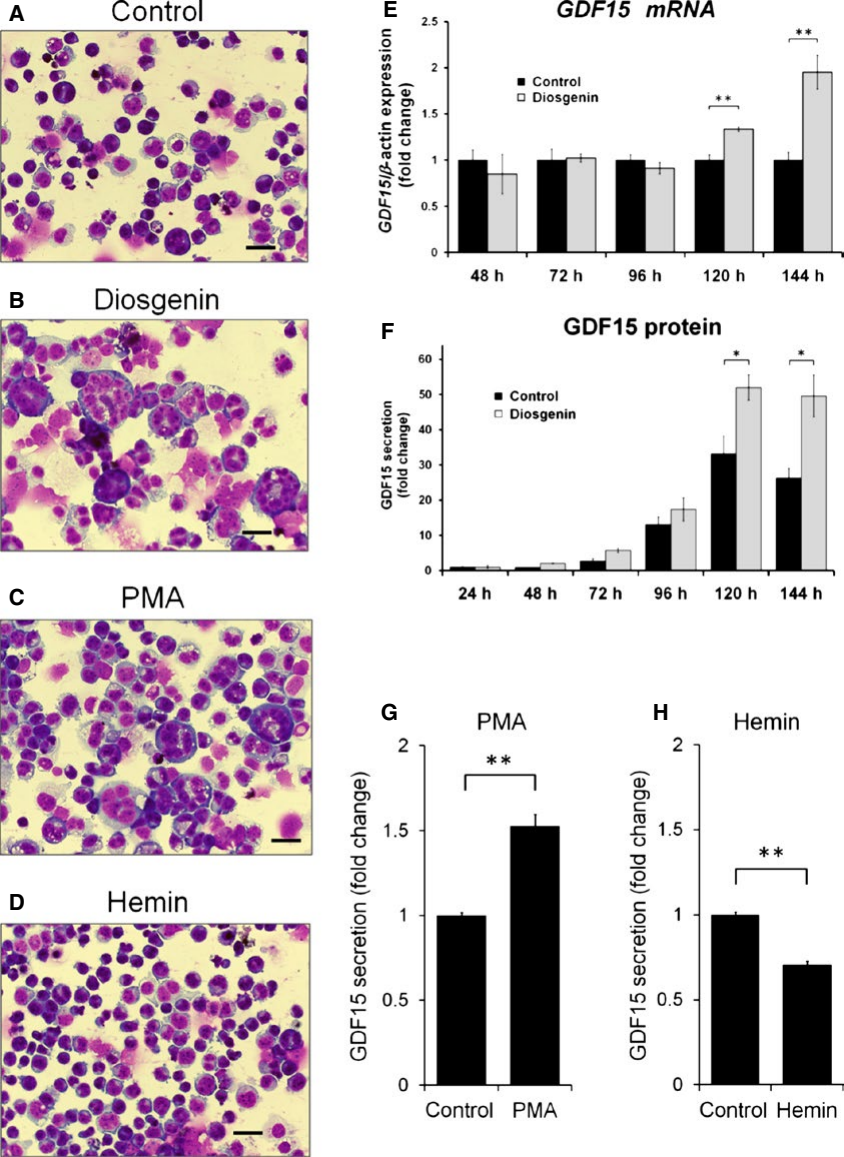
Expression of Growth differentiation factor 15 (GDF15) during megakaryocytic differentiation of HEL cells. (A−D) May-Grunwald-Giemsa staining (original magnification, ×400). HEL cells were cultured in the presence of (A) control solvent, (B) 10 *μ*mol/L diosgenin, (C) 10 nmol/L PMA, or (D) 50 *μ*mol/L hemin for 96 h. (E) Expression of GDF15 mRNA in HEL cells during megakaryocytic differentiation by diosgenin. Cells were treated with 10 *μ*mol/L diosgenin for 48, 72, 96, 120, and 144 h, and *GDF15*mRNA levels were measured by quantitative real-time PCR. The relative expression level of *GDF15* was normalized to that of *β-actin* and is shown relative to the expression levels of the control. (F–H) GDF15 protein secretion levels in the culture supernatant determined by ELISA. HEL cells were cultured in the presence of (F) 10 *μ*mol/L diosgenin for 24, 48, 72, 96, 120, and 144 h, (G) 10 nmol/L PMA for 96 h, or (H) 50 *μ*mol/L hemin for 96 h. Values represent the mean fold change compared to the respective control from triplicate experiments ± SE (*n* = 3). *P < 0.05 versus control; **P < 0.01 versus control.

Proliferation of megakaryocytes in the BM is one of the characteristic features of PMF. To investigate the expression of GDF15 in megakaryocytic cells in vitro, we used a human erythroleukemia cell line, HEL. This cell line differentiates toward the megakaryocytic lineage in response to diosgenin or PMA, and it differentiates toward the erythroid lineage in response to hemin 31–33. Consistent with megakaryocytic differentiation, the cell size increased, some of the cells were multinucleated (Fig.3A–D), and cell surface expression of CD235 decreased (Fig. S2) after 144 h of incubation with diosgenin or PMA. Cell surface expression of CD41, a megakaryocytic marker, slightly increased after incubation with diosgenin, and it clearly increased after incubation with PMA (Fig. S2).

We examined GDF15 mRNA expression and protein secretion during HEL cell differentiation. Quantitative real-time PCR revealed that GDF15 mRNA expression was significantly upregulated during diosgenin-induced differentiation toward the megakaryocytic lineage in comparison with untreated HEL cells (Fig.3E). Furthermore, the GDF15 concentration in the culture medium of diosgenin-stimulated HEL cells significantly and progressively increased from 120 to 144 h relative to that in the culture medium of unstimulated cells (Fig.3F). Similar results were obtained when we used PMA to induce megakaryocytic differentiation in these cells (Fig.3G). In contrast, the amount of GDF15 in the culture supernatants of HEL cells differentiated toward the erythroid lineage by hemin decreased on a per-cell basis as a result of an increase in cell number (Fig.3H). These results indicate that megakaryocytic HEL cells are able to synthesize GDF15.

### GDF15 enhances the capacity of BM-MSCs to support HPC proliferation

**Figure 4:**
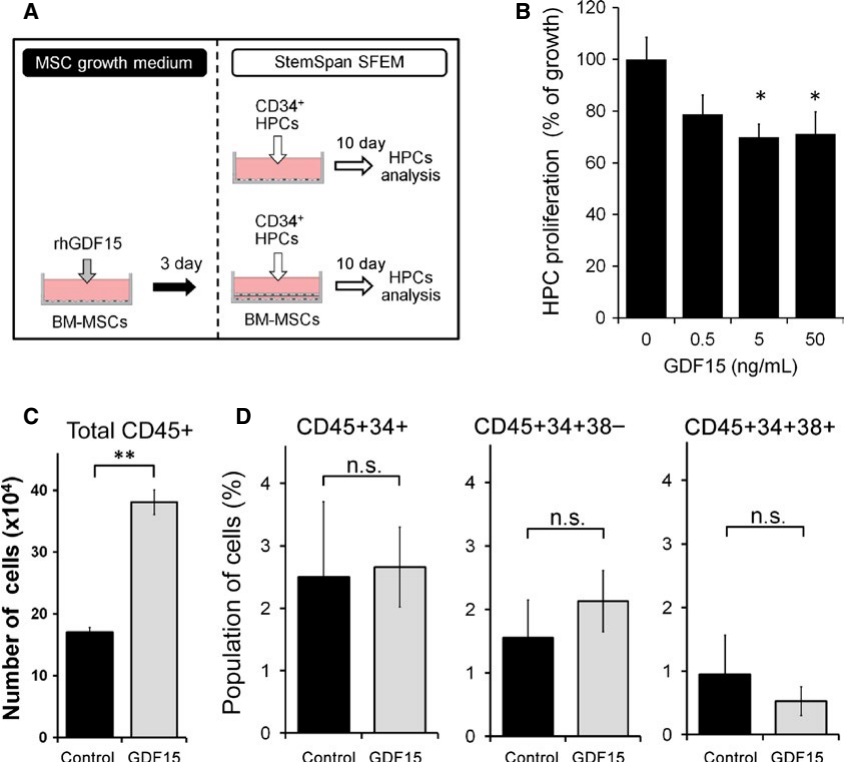
Effect of Growth differentiation factor 15 (GDF15) treatment of human BM-MSCs on expansion of HPCs. (A) Schematic representation of the experimental procedure used to analyze the effect of GDF15 on the proliferation of CD34^+^ HPCs cultured in the absence of human BM-MSCs as feeders (upper) and on the ability of human BM-MSCs to facilitate the expansion of CD34^+^ HPCs (lower). (B) Number of HPCs in the absence of feeder cells. HPCs were cultured in the absence or presence of 0.5, 5, and 50 ng/mL rhGDF15 for 10 days in the absence of feeder cells, and the cells were then counted in a hemocytometer by using Trypan blue exclusion. The results are presented relative to those of the untreated control. Each point signifies the mean from five replicates; bars indicate ± SE. (C) Number of total CD45^+^ hematopoietic cells expanded after 10 days of co-culture with either nonstimulated or rhGDF15 (30 ng/mL)-stimulated BM-MSCs, as determined by flow cytometry and a hemocytometer (*n* = 5). (D) Percentage of total CD45^+^CD34^+^ hematopoietic cells (left panel), and the ratios of CD38^–^ (middle panel) and CD38^+^ (right panel) cells among them, expanded after 10 d of co-culture with either nonstimulated or rhGDF15-stimulated BM-MSCs. SFEM, serum-free expansion medium. *P < 0.05 versus control; **P < 0.01 versus control; n.s., not significant.

Because GDF15 has been recognized as a stress cytokine that protects cells and tissues from various cellular stresses and has been reported to support the growth of various types of cells, we hypothesized that in PMF, GDF15 is produced to rescue the impaired BM environment to support hematopoiesis 9,10. To test this hypothesis, we examined the effect of rhGDF15 on the proliferation and differentiation of HPCs. As previously reported, CD34^+^ HPCs from human BM cannot efficiently proliferate in the absence of feeder cells, but they proliferate in the presence of human BM-MSCs as feeder cells 26,34,35. In the absence of these feeder cells, GDF15 did not support proliferation of HPCs, but rather suppressed their growth (Fig.4A upper panel, B). Next, we treated BM-MSCs with 30 ng/mL rhGDF15 for 3 days and used these cells as feeder cells in a direct contact co-culture system with HPCs. After 10 days of co-culture, adherent and nonadherent cells were collected, and the number of the cells was calculated using flow cytometry and a cell counter (Fig.4A lower panel). CD45^+^ total hematopoietic cells expanded more effectively when co-cultured with GDF15-pretreated human BM-MSCs than when co-cultured with untreated human BM-MSCs (Fig.4C). However, the ratios of total CD45^+^CD34^+^ HPCs, CD45^+^CD34^+^ CD38^−^ immature HPCs and CD45^+^CD34^+^CD38^+^ less immature HPCs among the total CD45^+^ cells were unchanged (Fig.4D). Taken together, these results indicate that GDF15-pretreated BM-MSCs possess the potential to support the proliferation of HSCs without affecting differentiation.

### GDF15 promotes human fibroblast proliferation

**Figure 5:**
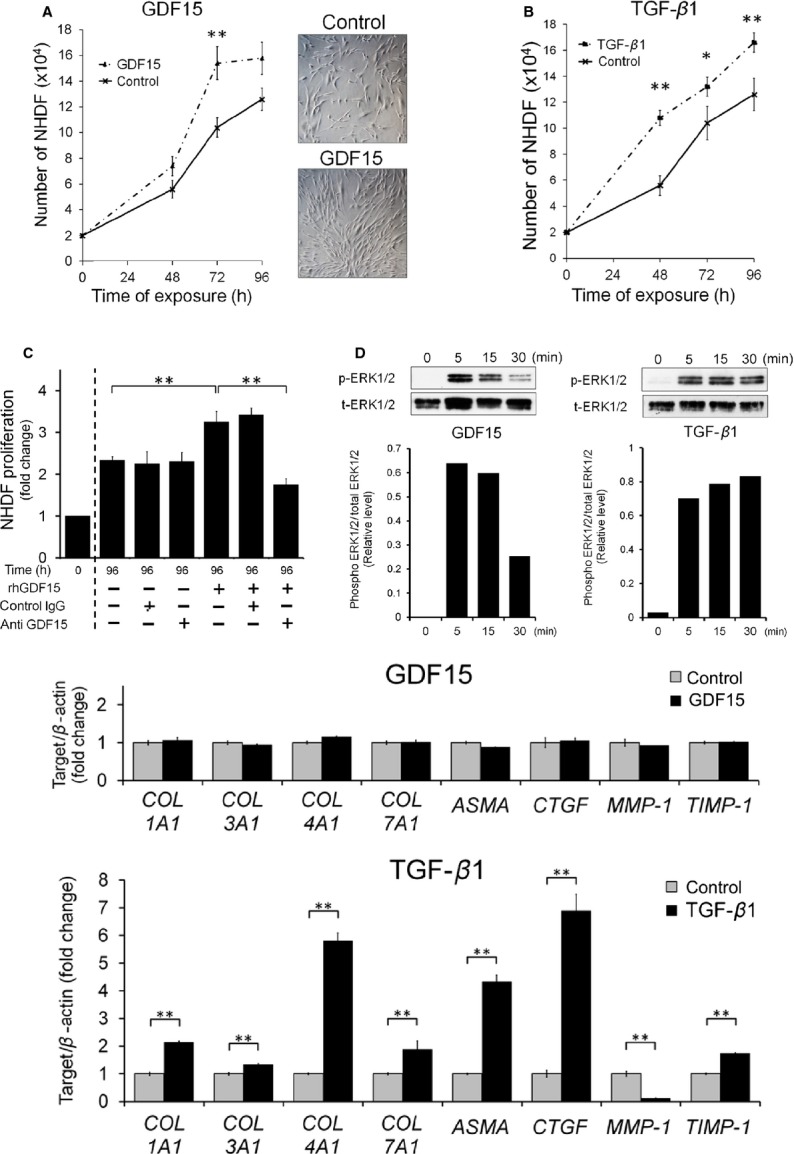
Effects of Growth differentiation factor 15 (GDF15) on normal human dermal fibroblasts (NHDFs). (A and B) Effects of rhGDF15 and rhTGF-*β*1 on proliferation of NHDFs. NHDFs were cultured in the absence or presence of (A) 30 ng/mL rhGDF15 or (B) 2 ng/mL rhTGF-*β*1 for 24, 48, 72, and 96 h, and the cells were counted with a hemocytometer using Trypan blue exclusion. Each point signifies the mean from five replicates; bars indicate ± SE (left panels). (A, right panels) NHDF were visualized using light microscopy after 72 h of rhGDF15 exposure (original magnification, ×400). (C) Effect of an anti-GDF15 neutralizing antibody on GDF15-induced NHDF proliferation. NHDFs were treated with rhGDF15 in the absence or presence of anti-GDF15 neutralizing antibodies (1 *μ*g/mL) or control isotype-matched mouse IgG for 96 h, and the cells were counted with a hemocytometer. (D) Immunoblot analysis of ERK1/2 phosphorylation in NHDFs treated with either rhGDF15 (left panels) or rhTGF-*β*1 (right panels) for 5, 15, and 30 min. The relative phosphorylation levels of ERK1/2 were normalized to the levels of total ERK1/2 protein and quantified using ImageJ. (E) Quantitative real-time polymerase chain reaction analysis examining extracellular matrix (ECM)-related mRNA expression in NHDFs treated with rhGDF15 or rhTGF-*β*1 for 3 days. The fold of increase in each mRNA in the treated group was obtained by normalization to untreated controls. COL1A1, collagen type 1 alpha 1; COL3A1, collagen type 3 alpha 1; COL4A1, collagen type 4 alpha 1; COL7A1, collagen type 7 alpha 1; ASMA, alpha-smooth muscle actin; CTGF, connective tissue growth factor; MMP-1, matrix metalloproteinase-1; TIMP-1, tissue inhibitor of metalloproteinase-1. Values represents the mean ± SE (*n* = 3). *P < 0.05 versus control; **P < 0.01 versus control.

Because the proliferation of fibroblasts in BM is one of the characteristic features of PMF, we evaluated the effect of GDF15 on fibroblasts. Normal human dermal fibroblasts (NHDF) were incubated with either rhGDF15 or rhTGF-*β*1, a well-known inducer of tissue fibrosis 36. With the addition of either rhGDF15 or rhTGF-*β*1, the number of NHDFs significantly increased relative to the number of unstimulated control cells (Fig.5A and B). A neutralizing antibody for GDF15 significantly reduced the effect of rhGDF15 on the proliferation of NHDFs, whereas isotype-matched mouse IgG antibodies did not influence NHDF growth (Fig.5C). Next, we focused on the mechanisms underlying the effect of GDF15 on NHDF proliferation. Because GDF15 is a TGF-*β* superfamily cytokine, we assumed that it might use the extracellular signal-regulated kinase (ERK), Akt, or Smad pathways. NHDFs were treated with either rhGDF15 or rhTGF-*β*1, and ERK1/2 phosphorylation was analyzed by immunoblotting. ERK1/2 was significantly phosphorylated by stimulation with GDF15 (Fig.5D). In contrast, the phosphorylation status of Akt remained unchanged by GDF15, and the total protein and phosphorylated-Smad3 levels were below the detection limit (data not shown). These results suggest that activation of ERK pathways may play a role in mediating the GDF15-induced proliferation of NHDFs.

Because TGF-*β*1 is known to participate in the process of fibrosis and pathogenesis of fibrotic disorders by affecting extracellular matrix (ECM)-related gene expression in fibroblasts, we determined whether GDF15 might have similar effects. Consistent with the results of previous reports, rhTGF-*β*1 induced mRNA expression of the profibrotic genes COL1A1, COL3A1, COL4A1, COL7A1, alpha-smooth muscle actin, connective tissue growth factor, and TIMP-1, and suppressed the expression of the antifibrotic MMP-1 gene in NHDFs 37–39. In contrast, rh GDF15 had no significant effect on ECM-related gene expression in NHDFs (Fig.5E). These results indicate that GDF15 promotes the proliferation of fibroblasts but does not affect the mRNA expression of ECM-related genes involved in the process of fibrosis.

### GDF15 promotes the proliferation and osteoblastic differentiation of human BM-MSCs

**Figure 6:**
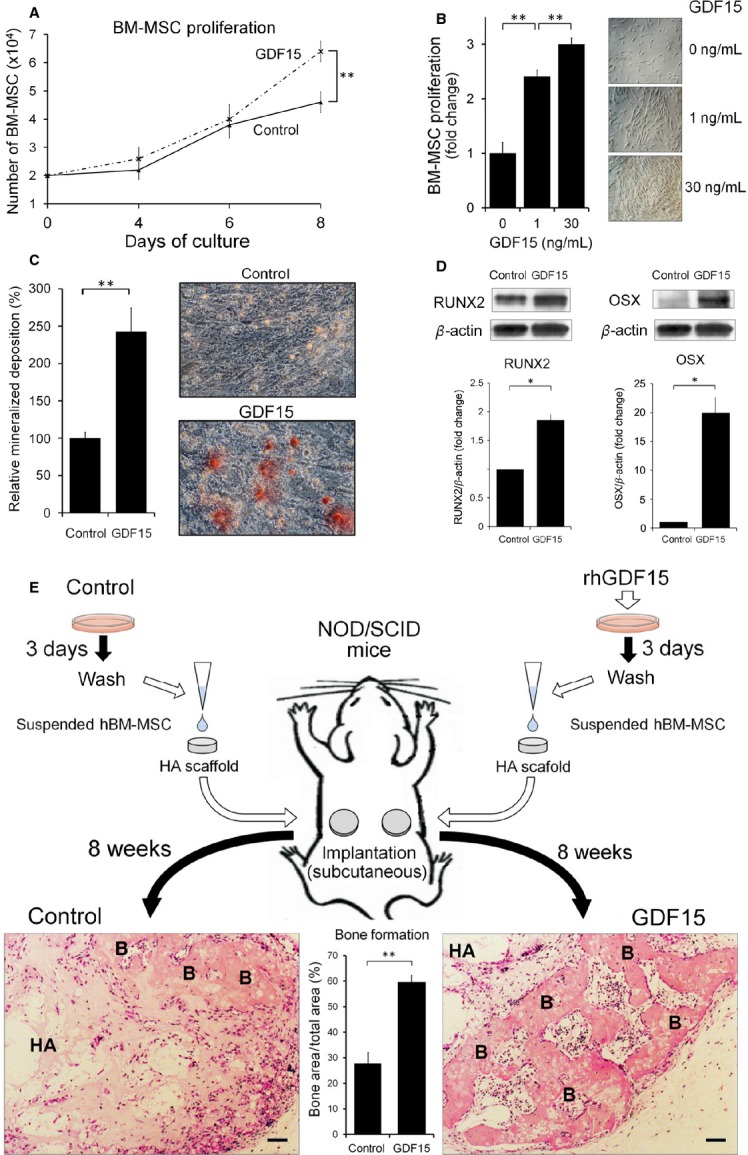
Effects of GDF15 on proliferation and osteoblastic differentiation of human bone marrow-derived mesenchymal stromal cells (BM-MSC). (A) Human BM-MSCs were cultured in the absence or presence of 30 ng/mL rhGDF15 for 4, 6, and 8 days, and the cells were counted in a hemocytometer. Each point signifies the mean from five replicates; bars indicate ± SE. (B) Human BM-MSCs cultured in the presence of 0, 1, and 30 ng/mL rhGDF15 for 8 days were counted in a hemocytometer. The results are presented relative to those of untreated controls (left panel), as visualized using light microscopy (right panels, original magnification ×400). Bars indicate ± SE. (C and D) GDF15-induced osteoblastic differentiation of BM-MSCs. (C) Human BM-MSCs were treated with rhGDF15 (30 ng/mL) for 4 weeks. Mineralized deposits indicated by Alizarin Red S staining were quantified using ImageJ in five different fields viewed at ×100 magnification. The results are presented relative to those of untreated controls (left panel), as visualized using light microscopy (right panels; magnification, ×100). (D) RUNX2 and osterix (OSX) protein expression in GDF15-treated BM-MSCs were analyzed by immunoblotting. The expression levels were normalized against *β*-actin protein levels and quantified using ImageJ. The results are presented relative to those of untreated controls. (E) In vivo bone formation assays. (Upper panel) Schematic representation of the experimental procedure used to analyze the effects of GDF15 on bone formation in a xenograft mouse model. Human BM-MSCs treated with rhGDF15 (30 ng/mL) for 3 days were subcutaneously transplanted into NOD/SCID mice (*n* = 4) using a hydroxyapatite-poly d,l-lactic-co-glycolic acid composite block type scaffold (HA scaffold) as a carrier and harvested 10 weeks later. (Lower right and left panels) Representative microscopic images and quantitative measurement of bone formation areas in sections of human BM-MSC implants stained with hematoxylin and eosin. HA, hydroxyapatite; B, bone. Bar = 50 *μ*m. The results are expressed as the ratio of the total bone area to the total implant area, quantified using ImageJ in five different fields viewed at ×100 magnification (lower middle panel). Bars indicate ± SE. *P < 0.05; **P < 0.01.

Because osteosclerosis is another characteristic feature of PMF and osteoblasts are derived from BM-MSCs, we next investigated the effects of GDF15 on the proliferation and differentiation of human BM-MSCs. In an in vitro culture system, the number of BM-MSCs significantly increased after 1 week in the presence of rhGDF15, and this effect was concentration-dependent (Fig.6A and B). We then investigated whether GDF15 promotes the differentiation of BM-MSCs into osteoblastic lineages. Human BM-MSCs exposed to rhGDF15 for 4 weeks exhibited positive Alizarin Red S staining, indicating mineralized deposits, whereas control human BM-MSCs exhibit a lack of clear staining, indicating that GDF15 had the capacity to induce bone formation (Fig.6C). Western blot analysis revealed that protein levels of Runx2 and osterix (OSX), representative markers of osteoblastic differentiation, were significantly elevated in rhGDF15-treated human BM-MSCs compared to control cells in this condition (Fig.6D). Next, we examined whether GDF15 affects the adipogenic differentiation of human BM-MSCs. Cells were cultured in the presence or absence of rhGDF15 for 2 weeks and stained with Oil Red O, which reflects adipogenetic differentiation. There were no significant differences in Oil Red O staining between GDF15-treated and untreated cells (data not shown).

These in vitro results prompted us to investigate the effect of GDF15 on bone formation in a xenograft mouse model that we previously established 24,26,27,29,40,41. Either GDF15 pretreated or untreated human BM-MSCs were seeded on HA scaffolds and subcutaneously implanted in NOD/SCID mice. After 10 weeks of implantation, the implants were histologically examined. The percentage of bone area relative to the total area of formed connective tissues in the HA scaffolds was significantly higher in GDF15-pretreated human BM-MSC implants than in untreated BM-MSC implants (Fig.6E). Taken together, these findings indicate that GDF15 has the potential to induce human BM-MSCs to differentiate toward the osteoblastic lineage both in vitro and in vivo.

## Discussion

In this study, we demonstrated that the concentration of serum GDF15 was significantly increased in patients with various hematologic malignancies in comparison with healthy controls. These results were consistent with those of previous reports showing elevated levels of serum GDF15 in PV, ET, and MM patients 18,19. Among the hematologic diseases that we evaluated, PMF showed particularly high serum GDF15 concentrations.

PMF, the most severe type of *BCR-ABL1*-negative MPN, is characterized by sustained proliferation of abnormal megakaryocytes, BM fibrosis, osteosclerosis, extramedullary hematopoiesis, splenomegaly, and anemia. These symptoms are progressive, and some patients eventually develop acute leukemia. The prognosis of patients with this disease is generally unfavorable 42. Blood cells of half of the patients carry *JAK2 V617F* mutant alleles, whereas mutant *MPL* genes are less common 43. Clonal expansion of abnormal megakaryocytes and stromal reactions caused by humoral factors released from these megakaryocytes, such as TGF-*β*, basic fibroblast growth factor (bFGF), and platelet-derived growth factor (PDGF), have been implicated in the pathogenesis of BM fibrosis in PMF 44. In addition, expression of BMPs, including BMP1, BMP6, and BMP7, is elevated in patients in the advanced stages of PMF, and megakaryocytes and BM-MSCs are considered to be the major sources of BMPs 45. In particular, enhanced expression of BMP6 was observed in the prefibrotic stage of PMF and was shown to be critical in the fibrotic switch from early to more advanced stages of PMF 45.

Among the cell types in BM, erythroid progenitors, macrophages, and MSCs have been shown to produce GDF15 14,21. In addition, our results for IHC staining indicated that megakaryocytes may be another source of GDF15 in BM. The possibility that megakaryocytes absorbed GDF15 from their environment and simply stored it in their cytoplasm could not be excluded; however, our in vitro HEL cell differentiation assay showed that megakaryocytic cells produced GDF15. The remarkably elevated serum concentrations of GDF15 in PMF patients observed in our study agree with the increased number of megakaryocytes observed in PMF.

GDF15 is regarded as a stress cytokine that protects cells from various types of damage such as hypoxia. Therefore, we initially hypothesized that in PMF, GDF15 is produced in response to impairment of the hematopoietic environment in BM to support hematopoiesis. Consistent with this hypothesis, human HPCs were more effectively expanded when co-cultured with human BM-MSCs that had been pretreated with GDF15 than when co-cultured with untreated BM-MSCs. Early-stage osteoblastic cells with high expression of Runx2 have been shown to possess strong potential to support HPC proliferation 46. The GDF15-induced osteoblastic differentiation that we showed in this study may thus enhance the hematopoiesis-supporting potential of human BM-MSCs.

Increased fibrous connective tissue levels, one of the characteristic features in PMF, are mainly caused by increased production of ECM including collagen and *α*-smooth muscle actin (*α*SMA). The amount of ECM is determined by not only the production of these fibrotic molecules but also the production of MMPs that dissolve the ECM and TIMPs that inhibit the activity of MMPs 47. Several studies have shown that TGF-*β* secreted from abnormal megakaryocytes activates fibroblasts, promotes ECM deposition, suppresses production of MMPs, and leads to BM fibrosis in PMF 39,48. Similarly, PDGF and bFGF have also been implicated in BM fibrosis through proliferation of fibroblasts and stromal cells and have been shown to support vascular endothelial cell growth 49. The results of this study demonstrated that GDF15 protein is highly expressed in megakaryocytes and enhances the proliferation of both fibroblasts and MSCs; however, in contrast to TGF-*β*, it does not alter the expression of ECM-related genes in vitro. Recently, Lambrecht et al. did not observe differences in the development of pulmonary fibrosis between wild-type and GDF15-deficient mice 50. Therefore, GDF15 may not play essential roles in the development of tissue fibrosis.

Osteosclerosis, a bone disorder characterized by abnormal bone thickening and increased bone mass, is another common pathological feature of PMF. Osteosclerosis occurs as a result of imbalances in bone remodeling, which is a process of bone formation by osteoblasts and bone resorption by osteoclasts. Osteoblasts are derived from mesenchymal progenitors, which are multipotent nonhematopoietic cells capable of differentiating into a variety of cell types, including osteoblasts, adipocytes, and chondrocytes, by the actions of growth factors produced locally or present in the circulation 51. Although GDF15 belongs to the BMP family, its role in bone remodeling is controversial. Subcutaneous implantation of Chinese hamster ovary cells that expressed recombinant GDF15 was shown to induce cartilage formation and the early stages of endochondral bone formation 5. In an in vitro experiment, GDF15 was reported to inhibit osteoclastic differentiation from RAW264.7 macrophages and BM precursors by M-CSF/RANKL in a concentration-dependent manner 52. In contrast, hypoxia-induced overexpression of GDF15 in mice was reported to promote osteoclastic differentiation, thereby reducing bone volume 53. Wakchoure et al. inoculated nude mice with DU-145 human prostatic cancer cells overexpressing GDF15 and observed both enhanced osteoblast differentiation and osteoclast numbers at sites of bone metastases 54. The results of our study indicate that exposure to very high concentrations of GDF15 induced mineral depositions and expression of the osteoblastic lineage markers Runx2 and osterix in BM-MSCs in vitro and that pretreatment of human BM-MSCs with rhGDF15 enhanced bone formation in a xenograft mouse model, indicating that GDF15 has the potential to promote osteoblastic differentiation under certain conditions.

Considering that GDF15 is produced in response to cellular stress and acts to protect cells and tissues in various situations, it is likely to be produced in response to the impairment of hematopoietic environment in PMF 55,56. In the early (prefibrotic and hypercellular) stages of PMF, GDF15 derived from megakaryocytes may support hematopoiesis by affecting BM-MSCs as a component of the BM niche. However, our results also indicate that GDF15 promotes the differentiation of BM-MSCs toward the osteoblastic lineage under certain conditions. Therefore, in advanced (fibrotic and ostesclerotic) stages, GDF15 may contribute to the development of BM fibrosis and osteosclerosis by accelerating the growth of fibroblasts and promoting the osteoblastic differentiation of BM-MSCs (Fig. S3).

In summary, we demonstrated that serum GDF15 concentrations in patients with PMF are remarkably elevated, and in BM, GDF15 is predominantly expressed by megakaryocytes. Our in vitro and in vivo studies suggest that GDF15 may be involved in the pathogenesis of PMF by promoting osteoblastic differentiation of BM-MSCs. Further studies are needed to assess the value of GDF15 as a new biomarker for evaluating treatment response and monitoring disease progression in hematologic malignancies including PMF, and to understand the precise roles of this cytokine in the BM microenvironment in both physiological and pathological conditions.

## Supporting Information

**Figure S1.** Histological examination of growth differentiation factor 15 (GDF15) expression in bone marrow (BM) clots without decalcification. (A) BM clot sections with normal morphology obtained from patients with localized gastric cancer. GDF15 is principally expressed by megakaryocytes. Original magnification, ×400.

**Figure S2.** In vitro differentiation of HEL cells. HEL cells were cultured in the presence of 10 *μ*mol/L diosgenin, 50 *μ*mol/L hemin, or 10 nmol/L PMA for 144 h, which was followed by flow-cytometric analyses of cell surface expression of CD41 and CD235a. PE-conjugated mouse anti-human CD41 (Cosmo Bio, Tokyo, Japan) and CD235a (BD Biosciences, Franklin Lakes, NJ) antibodies were used. Shaded purple areas represent histograms of isotype of control experiments.

**Figure S3.** A hypothetical scheme for the roles of GDF15 in primary myelofibrosis. GDF15 may be produced in response to the impairment of hematopoietic environment. In the early prefibrotic stage, GDF15 derived from megakaryocytes may support hematopoiesis by affecting BM-MSCs as a component of the BM niche. In the fibrotic stages, however, GDF15 may contribute to the development of BM fibrosis by accelerating the growth of fibroblasts together with TGF-*β*-induced ECM production. In a more advanced stage, excessive or prolonged GDF15 production may lead to the development of osteosclerosis by promoting osteoblastic differentiation of BM-MSCs. PMF, primary myelofibrosis; MK, Megakaryocyte; MSC, mesenchymal stroma cell; HPC, hematopoietic progenitor cell; GDF15, growth differentiation factor 15; TGF-*β* transforming growth factor, *β*-ECM, extracellular matrix; BM, bone marrow.

**Table S1.** Primer sequences of the target genes used in quantitative RT-PCR.

**Table S2.** Clinical features of patients with myeloproliferative neoplasms.
