# Supplementary figures and images for: The role of growth differentiation factor 15 in the pathogenesis of primary myelofibrosis
Source: Cancer Med. 2015 Aug 15;4(10):1558–72. doi: 10.1002/cam4.502 (PMC4618626; doi:10.1002/cam4.502)

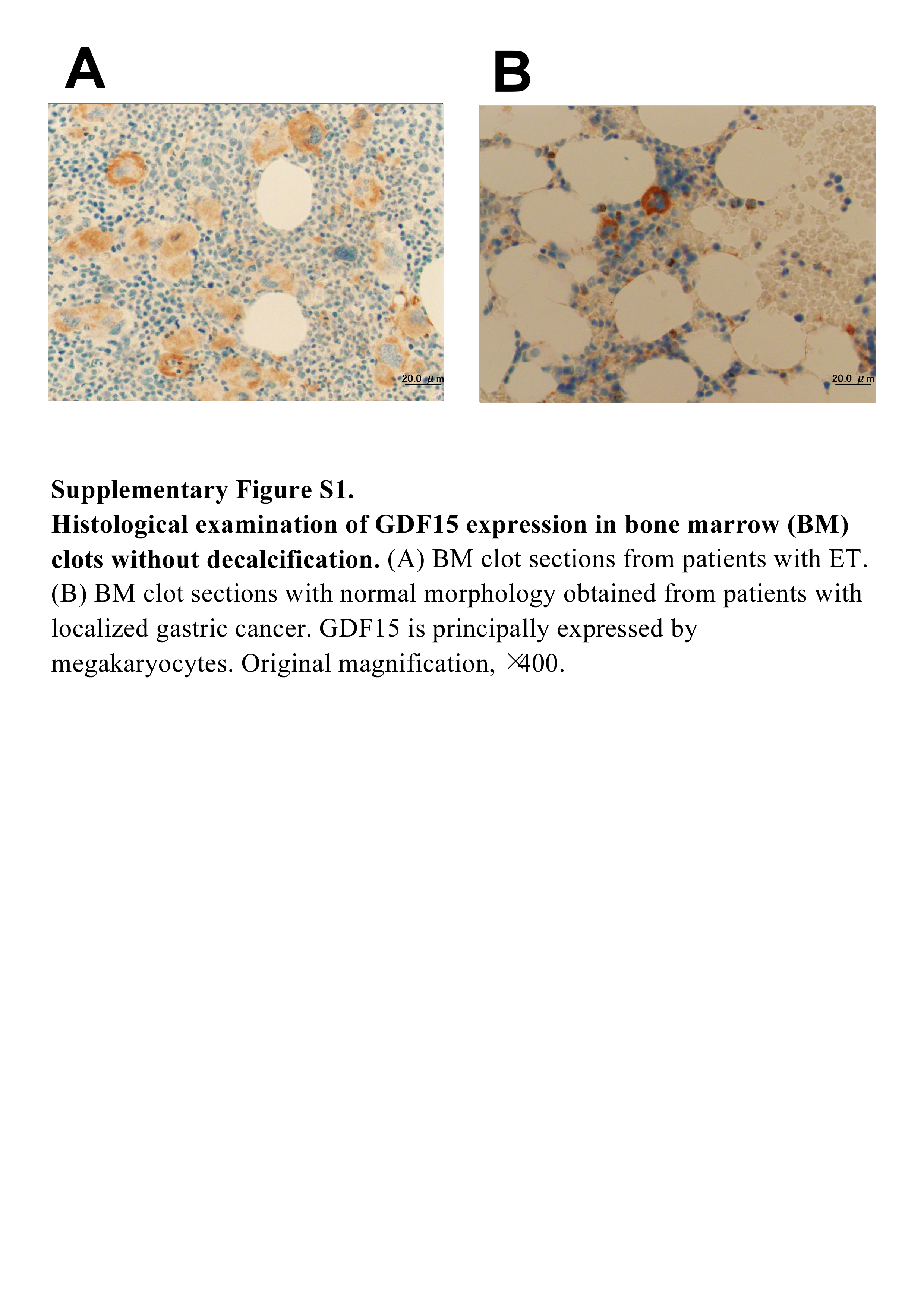

Supplement: Supplementary file 1 [file cam40004-1558-sd1.tif]

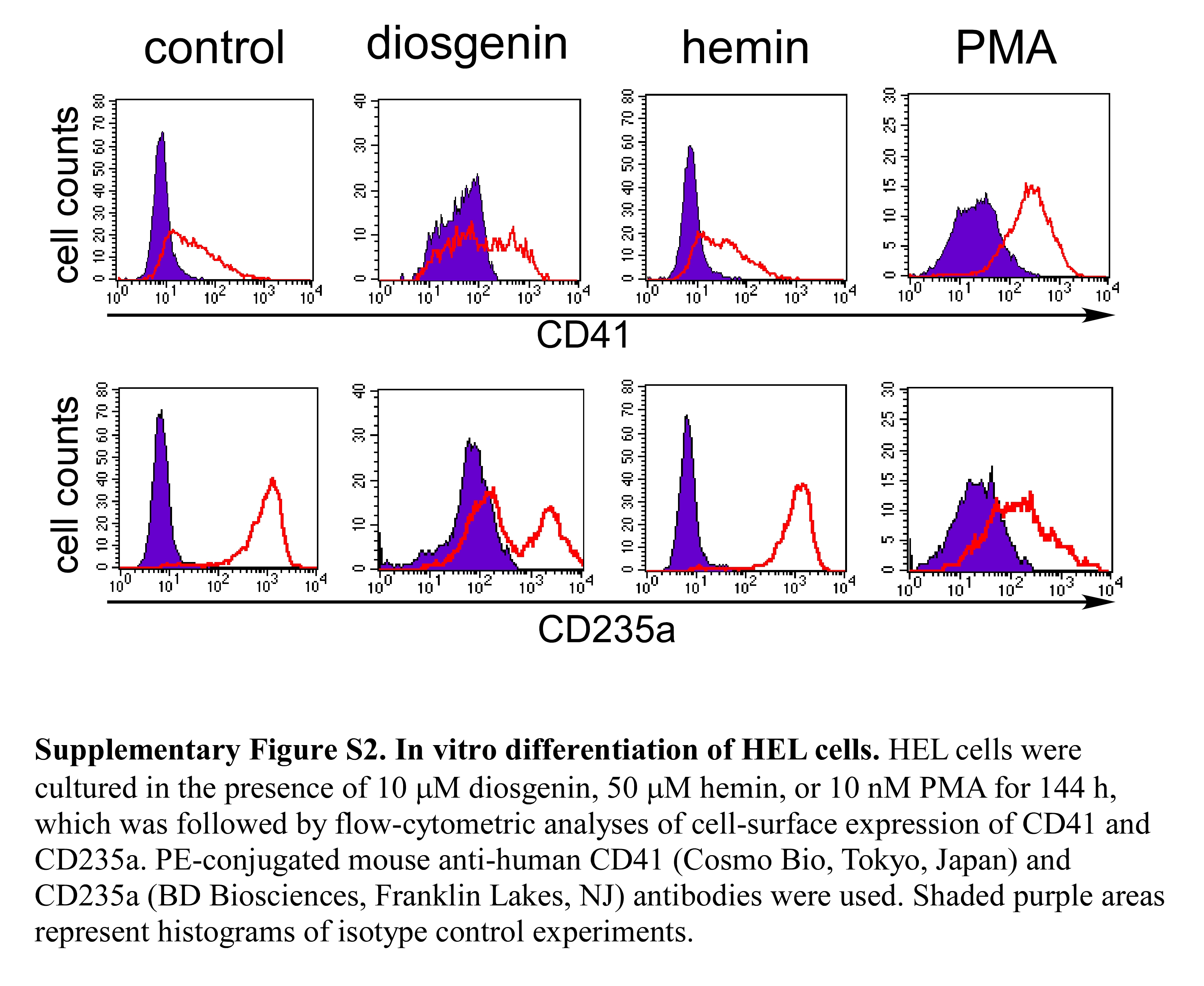

Supplement: Supplementary file 2 [file cam40004-1558-sd2.tif]

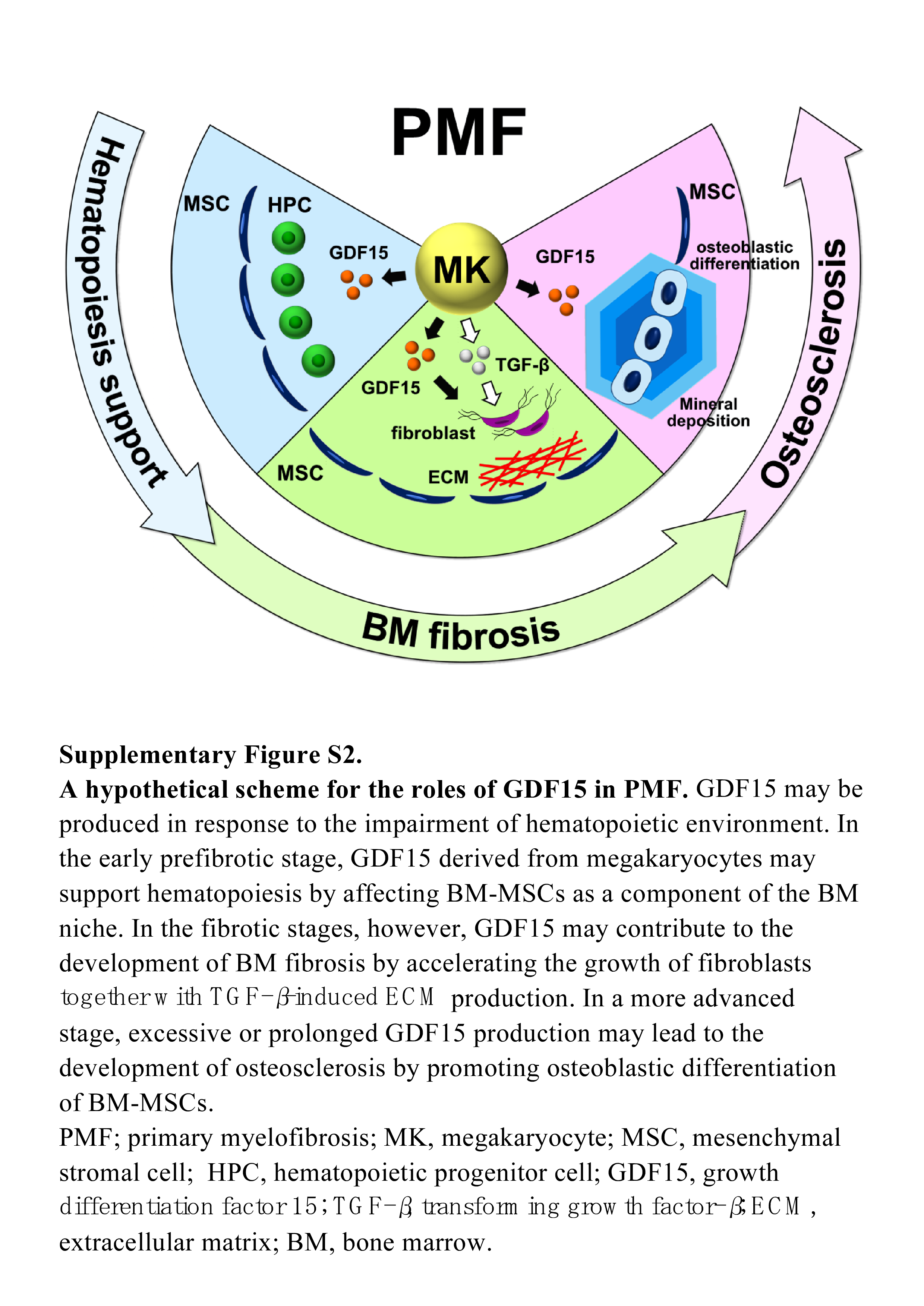

Supplement: Supplementary file 3 [file cam40004-1558-sd3.tif]
