# Supplementary material for: The role of growth differentiation factor 15 in the pathogenesis of primary myelofibrosis
Source: Cancer Med. 2015 Aug 15;4(10):1558–72. doi: 10.1002/cam4.502 (PMC4618626; doi:10.1002/cam4.502)
Supplement: Supplementary file 4 [file cam40004-1558-sd4.docx]

**Supplementary Table S1.**

**Primer sequences of the target genes used in quantitative RT-PCR**

| Target gene | | Primer nucleotide sequence |
| --- | --- | --- |
| β-actin | (forward) | 5ʹ-AGAGCTACGAGCTGCCTGAC-3ʹ |
|  | (reverse) | 5ʹ-AGCACTGTGTTGGCGTACAG-3ʹ |
| GDF15 | (forward) | 5ʹ-CTCCAGATTCCGAGAGTTGC-3ʹ |
|  | (reverse) | 5ʹ-AGAGATACGCAGGTGCAGGT-3ʹ |
| COL1A1 | (forward) | 5ʹ-GAACGCGTGTCATCCCTTGT-3ʹ |
|  | (reverse) | 5ʹ-GAACGAGGTAGTCTTTCAGCAACA-3ʹ |
| COL3A1 | (forward) | 5ʹ-AACACGCAAGGCTGTGAGACT-3ʹ |
|  | (reverse) | 5ʹ-GCCAACGTCCACACCAAATT-3ʹ |
| COL4A1 | (forward) | 5ʹ-ACTCTTTTGTGATGCACACCA-3ʹ |
|  | (reverse) | 5ʹ-AAGCTGTAAGCGTTTGCGTA-3ʹ |
| COL7A1 | (forward) | 5ʹ-TTACGCCGCTGACATTGTGTT-3ʹ |
|  | (reverse) | 5ʹ-ACCAGCCCTTCGAGAAAGC-3ʹ |
| ASMA | (forward) | 5ʹ-GACCGAATGCAGAAGGAGAT-3ʹ |
|  | (reverse) | 5ʹ-CCACCGATCCAGACAGAGTA-3ʹ |
| CTGF | (forward) | 5ʹ-TTTGGCCCAGACCCAACTAT-3ʹ |
|  | (reverse) | 5ʹ-GTGCAGCCAGAAAGCTCAAA-3ʹ |
| MMP-1 | (forward) | 5ʹ-GGCCCACAAACCCCAAAAG-3ʹ |
|  | (reverse) | 5ʹ-ATCTCTGTCGGCAAATTCGTAAGC-3ʹ |
| TIMP-1 | (forward) | 5ʹ-GGGACACCAGAAGTCAACCA-3ʹ |
|  | (reverse) | 5ʹ-GGCTTGGAACCCTTTATACATC-3ʹ |

GDF15; growth differentiation factor 15, COL1A1; collagen type 1 alpha 1, COL3A1; collagen type 3 alpha 1, COL4A1; collagen type 4 alpha 1, COL7A1; collagen type 7 alpha 1, ASMA; alpha-smooth muscle actin, CTGF; connective tissue growth factor, MMP-1; matrix metalloproteinase-1, TIMP-1; tissue inhibitor of metalloproteinase-1
