# Supplementary material for: The role of growth differentiation factor 15 in the pathogenesis of primary myelofibrosis
Source: Cancer Med. 2015 Aug 15;4(10):1558–72. doi: 10.1002/cam4.502 (PMC4618626; doi:10.1002/cam4.502)
Supplement: Supplementary file 5 [file cam40004-1558-sd5.docx]

**Supplementary Table S2**

**Clinical features of patients with myeloproliferative neoplasms**

| No. | Age  Sex | | Diagnosis | Disease duration (months) | GDF15 (pg/mL) | HGB (g/dL) | PLT (10^9^/L) | WBC (10^9^/L) | Splenomegaly | BM fibrosis | *JAK2 V617F* | Treatment |
| --- | --- | --- | --- | --- | --- | --- | --- | --- | --- | --- | --- | --- |
| 1 | 80 | M | PMF | 90 | 15,882 | 6.8 | 456 | 13.3 | +++ | 2 or 3 | N/A | 6-MP |
| 2 | 62 | M | PMF | 13 | 11,828 | 6.6 | 32 | 1.5 | +++ | + | + | None |
| 3 | 73 | F | PMF | 208 | 10,833 | 4.5 | 107 | 12.5 | +++ | 3 | - | MEL |
| 4 | 58 | F | PMF | 129 | 10,380 | 12.5 | 193 | 11.0 | +++ | + | - | HU |
| 5 | 92 | F | PMF | 60 | 4,942 | 11.0 | 440 | 17.1 | +++ | 1 | + | HU |
| 6 | 53 | M | PMF | 36 | 4,860 | 7.5 | 556 | 6.0 | +++ | 3 | + | None |
| 7 | 81 | F | PMF | 45 | 4,855 | 9.9 | 62 | 1.0 | +++ | 2 or 3 | N/A | THAL |
| 8 | 66 | M | PMF | 3 | 4,182 | 9.3 | 447 | 57.4 | +++ | 2 | + | None |
| 9 | 62 | F | PMF | 2 | 2,618 | 12.6 | 502 | 33.1 | +++ | 2 | + | None |
| 10 | 43 | F | PMF | 17 | 2,339 | 8.9 | 133 | 4.9 | +++ | 2 or 3 | - | None |
| 11 | 64 | F | PV | 132 | 4,968 | 6.1 | 14 | 0.5 | +++ | N/A | N/A | irradiation |
| 12 | 70 | M | PV | 204 | 3,725 | 4.7 | 99 | 73.6 | +++ | 2 | + | HU |
| 13 | 63 | M | PV | 222 | 3,349 | 13.1 | 130 | 16.9 | +++ | N/A | + | HU |
| 14 | 85 | F | PV | 161 | 2,777 | 10.3 | 259 | 14.3 | + | N/A | + | HU |
| 15 | 81 | M | PV | 304 | 2,518 | 14.8 | 98 | 4.9 | +++ | N/A | N/A | None |
| 16 | 59 | F | PV | 60 | 2,458 | 15.2 | 846 | 8.9 | + | N/A | + | None |
| 17 | 52 | M | PV | 40 | 2,236 | 14.3 | 371 | 7.3 | + | 0 | + | HU |
| 18 | 65 | M | PV | 42 | 1,578 | 11.4 | 313 | 10.9 | ++ | N/A | + | HU |
| 19 | 54 | M | PV | 102 | 1,555 | 14.7 | 443 | 22.5 | ++ | 0 | + | HU |
| 20 | 67 | M | PV | 244 | 1,431 | 13.4 | 534 | 10.5 | + | 0 | + | None |
| 21 | 65 | F | PV | 51 | 1,365 | 11.7 | 410 | 31.1 | N/A | 0 | + | None |
| 22 | 65 | F | PV | 27 | 1,080 | 15.6 | 373 | 12.2 | + | 0 | + | None |
| 23 | 70 | F | PV | 73 | 1,048 | 13.9 | 399 | 9.2 | + | 0 | + | HU |
| 24 | 79 | F | ET | 17 | 4,881 | 15.2 | 846 | 32.2 | - | N/A | + | MTX |
| 25 | 88 | F | ET | 73 | 4,759 | 11.5 | 702 | 4.7 | N/A | N/A | - | HU |
| 26 | 70 | M | ET | 183 | 3,725 | 6.3 | 85 | 64.9 | + | 2 | - | HU |
| 24 | 79 | M | ET | 76 | 3,196 | 15.2 | 472 | 17.2 | + | 1 | + | HU |
| 27 | 69 | M | ET | 6 | 2,808 | 14.7 | 697 | 14.7 | - | 0 | + | None |
| 28 | 41 | M | ET | 29 | 1,907 | 12.8 | 884 | 11.1 | - | N/A | + | None |
| 29 | 65 | F | ET | 14 | 1,659 | 12.8 | 1260 | 18.0 | + | 1 | + | None |
| 30 | 71 | F | ET | 14 | 1,391 | 12.6 | 1149 | 9.8 | - | N/A | - | None |
| 31 | 58 | F | ET | 57 | 1,352 | 11.7 | 481 | 45.0 | + | 1 | + | HU |

BM fibrosis was graded according to European consensus on grading bone marrow fibrosis [57]. Presence of fibrosis without grading information was indicated as “+.” Splenomegaly was graded as follows: +, mild splenomegaly, with a size smaller than that of half of the liver; ++, moderate splenomegaly, with a size larger than that of half of the liver but smaller than that of the liver; +++, severe splenomegaly with a size larger than that of the liver. Data for some cases were unavailable because of unlinked anonymization and not included in this table. Abbreviations; GDF15, growth differentiation factor 15; 6-MP, mercaptopurine; MEL, melphalan; HU, hydroxyurea; THAL, thalidomide; MTX, methotrexate; N/A, not available.
